# Silk Fibroin Promotes the Regeneration of Pancreatic β-Cells in the C57BL/KsJ-*Lepr^db/db^* Mouse

**DOI:** 10.3390/molecules25143259

**Published:** 2020-07-17

**Authors:** So-young Park, Boyoung Kim, Yun Kyung Lee, Sueun Lee, Jin Mi Chun, Jun-Gyo Suh, Jun Hong Park

**Affiliations:** 1 Department of Medical Genetics, College of Medicine, Hallym University, Chuncheon, Gangwon-do 24252, Korea; enivac82@gmail.com (S.-y.P.); bykim@hallym.ac.kr (B.K.); jgsuh@hallym.ac.kr (J.-G.S.); 2 Department of Internal Medicine, Seoul National University Bundang Hospital, Seongnam 13620, Korea; leeykyung@gmail.com; 3 Herbal Medicine Resources Research Center, Korea Institute of Oriental Medicine, Naju-si 58245, Korea; leese@kiom.re.kr (S.L.); jmchun@kiom.re.kr (J.M.C.)

**Keywords:** diabetes mellitus, diabetic mice, silk fibroin, insulin, β-cell

## Abstract

Diabetes mellitus is a chronic metabolic disease, and its progression leads to serious complications. Although various novel therapeutic approaches for diabetes mellitus have developed in the last three decades, its prevalence has been rising more rapidly worldwide. Silk-related materials have been used as anti-diabetic remedies in Oriental medicine and many studies have shown the effects of silk fibroin (SF) in both in vitro and in vivo models. In our previous works, we reported that hydrolyzed SF improved the survival of HIT-T15 cells under high glucose conditions and ameliorated diabetic dyslipidemia in a mouse model. However, we could not provide a precise molecular mechanism. To further evaluate the functions of hydrolyzed SF on the pancreatic β-cell, we investigated the effects of hydrolyzed SF on the pancreatic β-cell proliferation and regeneration in the mouse model. Hydrolyzed SF induced the expression of the proliferating cell nuclear antigen (PCNA) and reduced the apoptotic cell population in the pancreatic islets. Hydrolyzed SF treatment not only induced the expression of transcription factors involved in the pancreatic β-cell regeneration in RT-PCR results but also increased neurogenin3 and Neuro D protein levels in the pancreas of those in the group treated with hydrolyzed SF. In line with this, hydrolyzed SF treatment generated insulin mRNA expressing small cell colonies in the pancreas. Therefore, our results suggest that the administration of hydrolyzed SF increases the pancreatic β-cell proliferation and regeneration in C57BL/KsJ-Leprdb/db mice.

## 1. Introduction

Diabetes mellitus (DM) is a kind of metabolic disease, and its prevalence has steadily increased worldwide over in the last three decades [1]. DM is a burden on the public health care system as it increases public medical expenditure, particularly in developing countries. Based on insulin dependence, DM is classified as insulin-dependent diabetes mellitus (IDDM, type 1 DM, hereafter termed T1DM) and non-insulin-dependent diabetes mellitus (NIDDM, type 2 DM, hereafter termed T2DM). T1DM is primarily caused by genetic components, and its major cause is the autoimmune destruction of the pancreatic β-cells [2]. Pancreatic β-cells secrete insulin in response to increases in blood glucose levels. Insulin secreted by the pancreatic β-cells is an essential regulator of glucose metabolism; thus, insulin therapy could be the best solution for T1DM. However, T2DM is a more complex metabolic disorder that is caused by a sedentary lifestyle and obesity. T2DM is characterized by the malfunction, dedifferentiation, and death of the pancreatic β-cells, causing significant increases in the risk of cardiovascular disease, renal dysfunction, retinopathy, and neuropathy [3,4].

In the past three decades, scientists around the world have made remarkable progress in understanding pathology, diagnosis, and treatment of T2DM. However, anti-diabetes drugs often cause side effects such as nausea, low glucose levels, weight gain, and bloating. Furthermore, long-term use of anti-diabetes drugs causes drug resistance, which limits the duration of anti-diabetes drug administration and requires combination therapy. While various T2DM clinical treatments can improve the blood glucose levels and prolong patient survival, the efficacy of the anti-diabetic drugs is still limited. One of the effective anti-diabetic therapeutic approaches is to increase or replace the lost pancreatic β-cells by regeneration.

Owing to its verified active ingredients and minimal side effects, a compound used in Oriental medicine has been suggested as alternative medicine in the treatment of T2DM. For example, the leaves, dried plants, roots, fruits, animal, and insect-derived products have been used for anti-diabetic remedies in Oriental medicine. Notably, silk-related materials, including silkworm and cocoon extracts, have been used for treating T2DM [5,6,7,8]. Silk fibroin (SF) is a major component of silk, and SF is a core silk protein composed of 18 different kinds of natural amino acids. SF has a molecular weight of 3.5–3.6 × 10^5^ Da. Hydrolyzed SF contains glycine (40%), alanine (30%), serine (10%), and tyrosine (10%) as the major amino acids [9]. Previously, we demonstrated that hydrolyzed SF showed protective effects on pancreatic β-cell against the hyperglycemic conditions both in in vitro and in vivo systems [9,10,11]. Recent papers showed that treatment of SF improved the blood glucose levels in the mouse model or insulin sensitivity in 3T3-L1 adipocyte [12,13]. Furthermore, Park et al. demonstrated that dietary consumption of transgenic rice which expressed spider SF can regulate blood glucose levels in the diabetic mouse model. This study suggest that SF has similar effects on blood glucose level regardless of insect species. [14]. However, the mechanisms of anti-diabetic effects and biological functions of SF on the pancreatic β-cells remain unclear.

Hydrolyzed SF induced the expression of insulin-like growth factor-1 (IGF-1) and reduced the generation of cellular reactive oxygen species (ROS) [10,15]. Moreover, hydrolyzed SF helped to maintain the pancreatic β-cell mass and ameliorated diabetic dyslipidemia in type 2 diabetic mouse model [9]. Recently, studies have suggested that hydrolyzed SF could regulate transcription factors, which are involved in cellular regeneration or survival [15,16,17]. Taken together, these results suggested that hydrolyzed SF could protect the pancreatic β-cell from diabetic conditions and might induce regeneration of the pancreatic β-cells. However, the detailed mechanism by which hydrolyzed SF increases pancreatic β-cell mass has not been explored in the mouse model. Therefore, in the present study, we examined the effects of hydrolyzed SF in the pancreatic β-cell in C57BL/KsJ-*Lepr^db/db^* (*db/db*) mice, a well-established in vivo type 2 diabetes model.

## 2. Results

### 2.1. Treatment with Hydrolyzed Silk Fibroin Increases the Pancreatic β-Cell Mass and Function in the db/db Mouse Model

**Figure 1:**
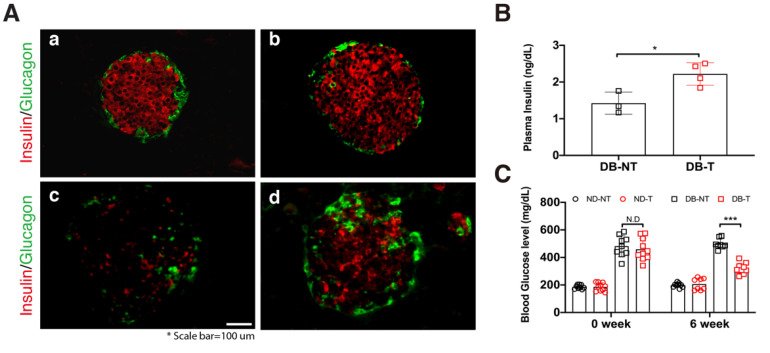
Effect of hydrolyzed silk fibroin (SF) on the pancreatic β-cells and blood glucose metabolism in the mouse model. (**A**) Immunofluorescence staining of the pancreatic islets in the normal (a: untreated non-diabetic mice (ND-NT), b: non-diabetic with hydrolyzed SF treatment (ND-T)), and diabetic (c: untreated *db/db* (DB-NT), d: hydrolyzed SF-treated *db/db* (DB-T)) mice. Slides were stained with *Insulin* (Alexa 594) and *Glucagon* (Alexa 488). Scale bar is indicated. (**B**) The plasma insulin levels in the *db/db* mice following 6 weeks of hydrolyzed SF treatment or not (*n* > 3). Data are represented as mean ± SEM. Student’s *t*-test, two-tailed, * *p* < 0.05. (**C**) The blood glucose levels in each experimental group following 6 weeks of hydrolyzed SF treatment or not (*n* > 7). Data are presented as mean ± standard error of the mean (SEM). One-way ANOVA, *** *p* < 0.001.

In our previous studies, we demonstrated that hydrolyzed SF treatment had protective effects against high glucose in the in vitro system [10] and ameliorated diabetic dyslipidemia in the *db/db* mouse model [11]. To confirm the effects of hydrolyzed SF on pancreatic β-cell numbers in the pancreatic islets, we administered hydrolyzed SF for 6 weeks via drinking water. After 6 weeks of hydrolyzed SF treatment, we conducted histological analysis using immunofluorescence staining in untreated non-diabetic mice (ND-NT), non-diabetic mice with hydrolyzed SF treatment (ND-T), untreated *db/db* mice (DB-NT), and hydrolyzed SF-treated *db/db* mice (DB-T) (Figure 1A). The pancreatic β-cell numbers increased in the DB-T group as compared with that in the DB-NT. Consistent with the results of histological analysis, the blood insulin levels increased in the DB-T group as compared with that in the DB-NT (Figure 1B). Supporting these results, blood glucose concentration was significantly decreased in the DB-T group following 6 weeks of the hydrolyzed SF treatment (Figure 1C). However, the change in body weight did not show a remarkable difference in each pair group (Table S1). These results suggested that the treatment of hydrolyzed SF increased the pancreatic β-cell numbers and enhanced the endocrine function.

### 2.2. Hydrolyzed Silk Fibroin Induces the Proliferation of Pancreatic β-Cells and Reduces Their Cellular Death

**Figure 2:**
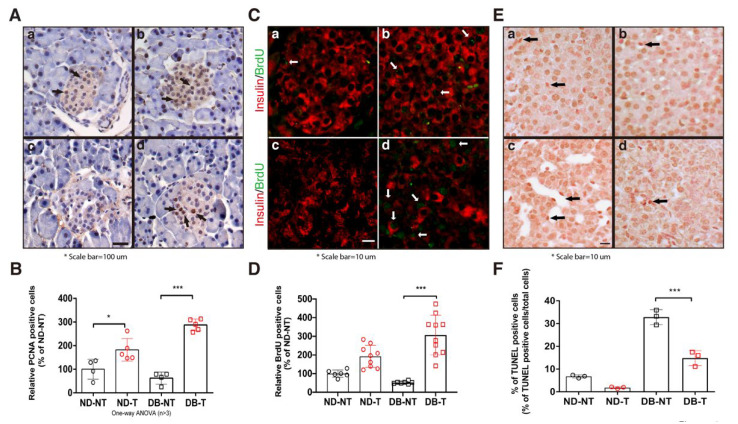
Effect of hydrolyzed SF on pancreatic β-cell proliferation and death in the pancreatic tissue. (**A**) Immunohistochemistry of pancreatic islets with antibody against the proliferating cell nuclear antigen (PCNA). Arrows point to representative PCNA-positive nuclei. (**a**: ND-NT, b: ND-T, **c**: DB-NT, and **d**: DB-T) (**B**) Quantification of PCNA-positive pancreatic β-cells (*n* > 4). Data are presented as mean ± SEM. One-way ANOVA, * *p* < 0.05, *** *p* < 0.001. (**C**) BrdU staining of pancreatic islets with the anti-BrdU antibody. White arrows indicated representative BrdU-positive signals. (**a**: ND-NT, **b**: ND-T, **c**: DB-NT, and **d**: DB-T) (**D**) Quantification of BrdU-positive pancreatic β-cells (*n* > 6). Data are presented as mean ± SEM. One-way ANOVA, *** *p* < 0.001. (**E**) TUNEL assay using the pancreatic islets from each experimental group. Arrows point to representative TUNEL-positive nucleus. (**F**) Quantification of percent of TUNEL-positive pancreatic β-cells (% of TUNEL positive cells/ Total cells) (*n* > 3). Data are presented as mean ± SEM. One-way ANOVA, *** *p* < 0.001.

In our previous report, hydrolyzed SF treatment induced the expression of proliferating cell nuclear antigen (PCNA) under high glucose conditions in HIT-T15 cells [10]. To confirm these results in the mouse model, we determined the number of proliferative and apoptotic cells in the pancreatic islets following hydrolyzed SF treatment. Hydrolyzed SF treatment increased the number of PCNA positive cells in the pancreatic islets in both the ND-T and DB-T groups (Figure 2A,B). To confirm the number of proliferative cells in the pancreatic islet, we performed the 5-Bromo-2’-Deoxyuridine (BrdU) assay. We used co-immunofluorescence staining with BrdU and insulin antibody for quantification of the BrdU-labeling pancreatic β-cell in the pancreas islet. We counted only the BrdU positive cells with insulin-positive cells, excluding the dual-color (yellow) signals. Consistent with the PCNA staining results, hydrolyzed SF treatment significantly increased the ratio of the BrdU positive cells in the pancreatic islets of the DB-T group as compared to that of the DB-NT group (Figure 2C,D). In the TdT-mediated dUTP nick end labeling (TUNEL) assay, we found evidence of reduced apoptosis by analyzing the sections of pancreatic islets derived from each group. Hydrolyzed SF treatment reduced the number of apoptotic cells in the pancreatic islets of the *db/db* mice (Figure 2E,F). These results demonstrated that hydrolyzed SF treatment induced the pancreatic β-cell proliferation and protected from the cellular death in the diabetic condition.

### 2.3. Hydrolyzed Silk Fibroin Induces the Expression of Transcriptional Factors Involved in the Regeneration of Pancreatic β-Cell

**Figure 3:**
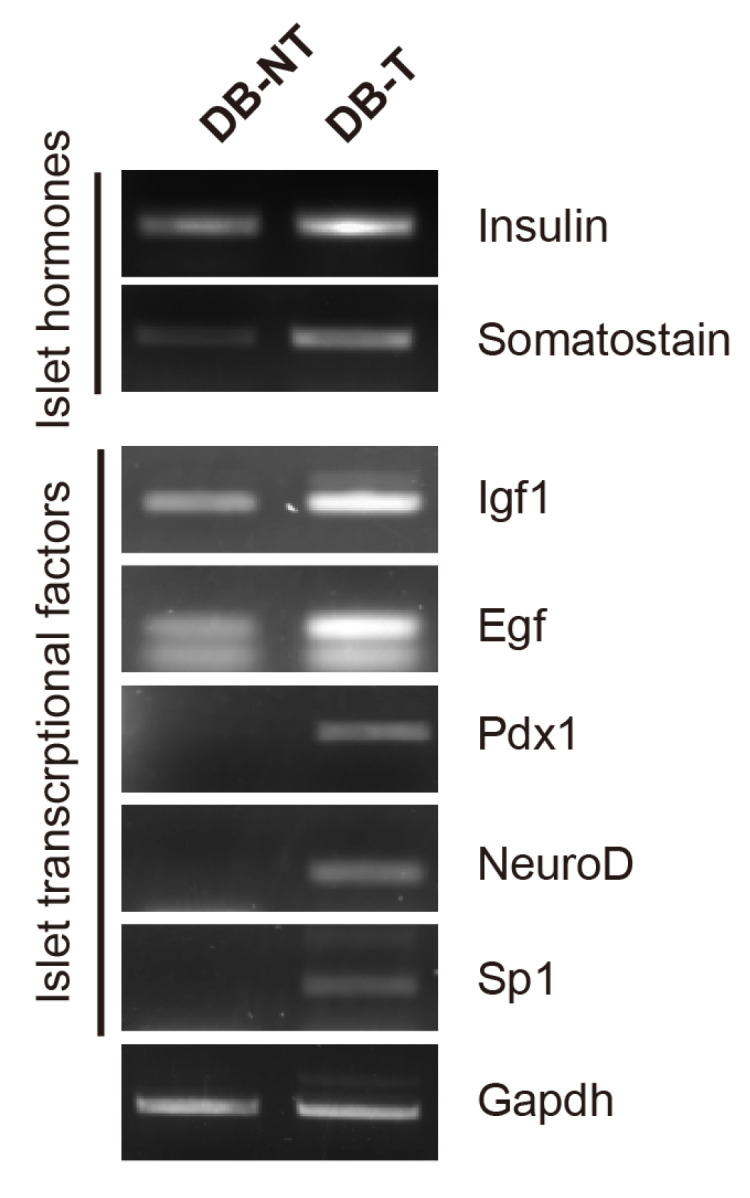
Effect of hydrolyzed SF on the transcriptional factors in the pancreatic islets. RT-PCR analysis of indicated transcriptional factors was performed in isolated pancreatic islets at 6 weeks after hydrolyzed SF treatment or not (DB-NT: *db/db* SF non-treated, DB-T: *db/db* SF treated).

A recent study demonstrated that hydrolyzed SF regulates the transcription factors, which involved in the tissue regeneration [16,17]; thus, we investigated whether hydrolyzed SF treatment can promote the regeneration of pancreatic β-cells in a mouse model. To confirm the regeneration of pancreatic β-cells, we compared the expression levels of transcriptional factors that are involved in the pancreatic β-cell regeneration in isolated pancreatic islets using reverse transcription polymerase chain reaction (RT-PCR) (Figure 3). Consistent with the blood insulin and glucose analysis, the hydrolyzed SF treatment increased the gene expression levels of insulin and somatostatin in the DB-T group. The gene expression levels of the transcriptional factors including insulin-like growth factor 1 (Igf1), epidermal growth factor (Egf), pancreas/ duodenum homeobox protein 1 (Pdx1), Neuro D, and Sp1 increased in the DB-T group. Since Pdx1 and Neuro D are essential regulators of the early development of the pancreatic β-cells, we investigated whether hydrolyzed SF treatment could affect the early pancreatic β-cell development markers in the mouse model. We performed Western blotting to examine if the increase in the gene expression of the transcriptional factors observed in the DB-T group could lead to an increase at the protein level (Figure S1). To clearly compare the protein expression levels between the DB-NT with the DB-T group, we loaded the DB-NT sample in the first lane. Consistent with the gene expression results, the protein expression levels of insulin increased in hydrolyzed SF-treated mice pancreatic islets, whereas the protein expression levels of glucagon had no difference in each pair group. Furthermore, we found that the protein expression levels of Ngn3, which is essential for pancreatic β-cell differentiation at an early stage, was slightly increased by hydrolyzed SF treatment in the DB-T group when compared to the DB-NT group.

**Figure 4:**
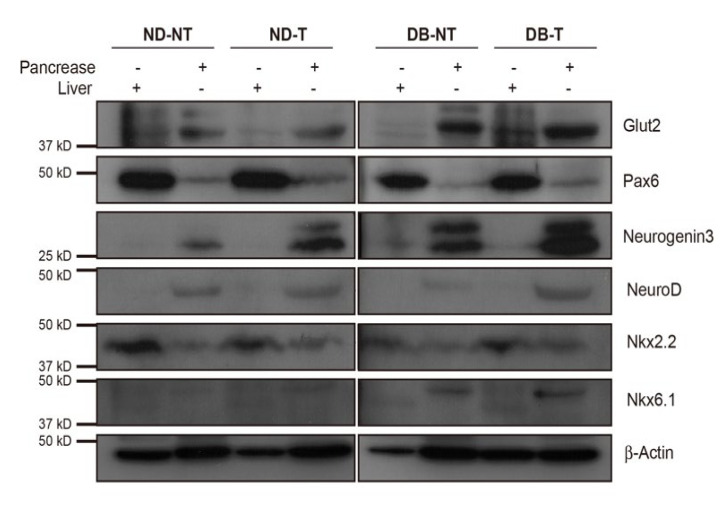
Hydrolyzed SF leads to improvement in the expression of pancreatic β-cell regeneration genes in the whole pancreatic tissue. Western blot analysis of the whole pancreas and liver tissue extracts with antibodies against Glut2, Pax6, neurogenin 3, Neuro D, Nkx2.2, Nkx6.1, and actin (ND-T: *db/+* SF treated, ND-NT: *db/+* SF non-treated, DB-T: *db/db* SF treated, and DB-NT: *db/db* SF non-treated).

As hepatocytes can undergo trans-differentiation into mature pancreatic β-cells or secrete important proteins that can regulate the pancreatic β-cell proliferation and development [18,19], we compared the expression levels of the transcriptional factors involved in the pancreatic β-cell development in the whole pancreas and liver tissues (Figure 4). Consistent with the gene and protein expression profiles of the pancreatic islets, the expression of Ngn3 in the whole pancreas increased in response to hydrolyzed SF treatment in the diabetic groups. Neuro D was increased in the pancreas of DB-T group mice compared to that of the DB-NT group. In the liver, the transcriptional factors examined did not show a remarkable difference between the diabetic condition and hydrolyzed SF treatment. These results suggested that the hydrolyzed SF treatment could increase not only the expression of the differentiation marker genes involved in the early development of the pancreatic β-cells, but also these modifications of gene expression might induce the regeneration of the pancreatic β-cells.

### 2.4. Hhydrolyzed Silk Fibroin Promotes the Regeneration of Pancreatic β-Cells

**Figure 5:**
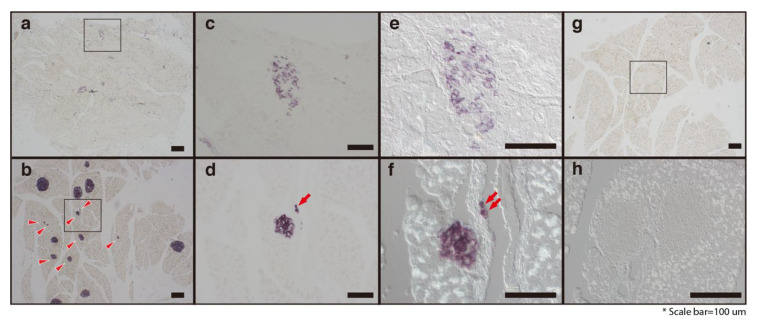
Treatment of hydrolyzed SF leads to the regeneration of pancreatic β-cells. DB-NT (**a**,**c**,**e**) and DB-T (**b**,**d**,**f**–**h**) pancreatic tissues were harvested and paraffin-embedded for RNA in situ analysis with antisense mouse insulin probe (**a**–**f**) or not (**g** and **h**, negative control). Panels (**c**) and (**d**) show higher magnification images of the boxed areas (**a**,**b**). Differential interference contrast (DIC) microscopy images of pancreatic islets from the DB-NT (**e**) and DB-T (**f**,**h**) groups. Red arrowheads point to the pancreatic islets that have a small size (**b**) and arrows indicate the neogenesis of the pancreatic β-cell (**d**,**f**). Scale bars are indicated.

To determine if hydrolyzed SF treatment could induce the pancreatic β-cell regeneration, we performed in situ hybridization (ISH) assays (Figure 5). Consistent with our RT-PCR and Western blotting results, hydrolyzed SF treatment increased the number of insulin-positive cells that strongly expressed the insulin mRNA in the pancreatic islets. The pancreatic islets in the mice in the DB-NT group contained fewer pancreatic β-cells, with faintly expressing insulin mRNA (Figure 5a,c). Furthermore, the hydrolyzed SF treatment increased the population of small colonies that expressed insulin mRNA in the pancreas (Figure 5b, arrowheads). Interestingly, the hydrolyzed SF treatment promoted the generation of insulin-positive cells around the pancreatic duct (Figure 5d,f). To confirm the morphological features of these cells, we used the differential interference contrast (DIC) microscopy imaging, a method that captures images with the appearance of a three-dimensional object (Figure 5e,f,h). Notably, DIC microscopy imaging of the pancreatic tissues from the DB-T mice showed that the ISH-positive cells can form a small colony (composed only of few positive cells) and are located on the pancreatic ductal epithelium (Figure 5f). We could not find cells with ISH positive signals in the negative control (Figure 5g,h). These results directly demonstrate that hydrolyzed SF treatment can promote not only the regeneration of the pancreatic β-cell but also the neogenesis of pancreatic islets around the pancreatic ducts in the mouse model.

## 3. Discussion

The silkworm and silkworm-derived natural products have been used as medicine for diabetes in Oriental medicine since ancient times [7,8]. However, the precise mechanism involved in the anti-diabetic effects of silk-related products remains unclear. In this study, we investigated the detailed mechanism involved in hydrolyzed SF treatment-mediated anti-diabetic effects and induction of the regeneration of the pancreatic β-cell in the diabetic *db/db* mouse model. Our data suggested that SF treatment increased the expression levels of transcriptional factors involved in the development of pancreatic β-cells, leading to their regeneration.

It has been reported that a high protein diet has a beneficial effect on blood glucose levels in T2DM [20,21]. As a natural resource of amino acids, hydrolyzed SF may promote insulin secretion and control glucose levels in diabetic patients. Additionally, the hydrolyzed SF used in this study was an acid hydrolyzed and freeze-dried digestible powder. Therefore, it may contain a mixture of variously sized peptides and individual amino acids. There is a possibility that these mixtures could affect the blood insulin and glucose levels. However, our previous study showed that the treatment with mimic amino acid mixtures, which have a similar composition of major amino acids like hydrolyzed SF, had no effects on blood glucose levels in the *db/db* mouse model [9]. These findings indicate that hydrolyzed SF probably helps to supplement the amino acids or proteins in the T2DM model mice, but promoting the regeneration of pancreatic β-cell is the direct anti-diabetic effect of hydrolyzed SF.

We have confirmed that the hydrolyzed SF treatment of the mouse model increased the pancreatic β-cell numbers and their function when compared to that of the untreated diabetic mice, as shown by our immunohistochemistry and blood analysis parameters. Natural compounds are novel resources for anti-diabetic drug development [22]. Recently, numerous natural compounds have demonstrated anti-diabetic potential through the regulation of pancreatic β-cell regeneration [23,24]. We could not directly compare the efficacy of hydrolyzed SF activity on promoting the regeneration of the pancreatic β-cell with the other natural compounds. When indirectly comparing the activity using the histological analysis, the efficacy of hydrolyzed SF might be expected to be similar or higher than the other natural compounds [25,26,27,28]. In our RT-PCR results, it was shown that the hydrolyzed SF treatment increased the gene expression of somatostatin in isolated pancreatic islets; these results suggest that hydrolyzed SF might promote not only the regeneration of pancreatic β-cell but also that of other endocrine cells in the pancreatic islets. The regeneration of the pancreatic β-cells can be suggested as one of the truly therapeutic ways to treat T2DM. Since Ballinger et al. showed that the transplantation of isogeneic islets can cure diabetes in the animal model [29], many clinicians and researchers have focused on the regeneration of the pancreatic β-cell for diabetes. The regeneration of pancreatic β-cells is regulated by many intrinsic and extrinsic factors. Our data show that the increase in the pancreatic β-cells might be due to 1) the direct role of hydrolyzed SF in inducing the regeneration of β-cells or 2) the indirect function of hydrolyzed SF in preventing the cell death caused by cellular stress. For example, hydrolyzed SF treatment reduced the generation of ROS and protected against the external stress [15,30]. We cannot pinpoint the precise mechanism responsible for the original function of hydrolyzed SF in these possibilities; however, our histology results strongly suggest a direct role of hydrolyzed SF in the regeneration of the pancreatic β-cell in the mouse model.

Although we could not detect a remarkable difference in protein analysis using isolated pancreatic islets, we found that the expression of Ngn3, which is involved in the pancreatic β-cell regeneration, was slightly increased by hydrolyzed SF treatment in the DB-T group when compared to the DB-NT group (Figure S1). In the analysis of protein expression levels using the whole pancreas, the diabetic conditions or hydrolyzed SF treatment (DB-NT or ND-T) slightly increased the protein expression levels of Ngn3 as compared to those of the ND-NT group (Figure 4). In addition, the Neuro D expression was increased in the DB-T group as compared to the DB-NT group (Figure 4). These data suggested that hydrolyzed SF may have a role in driving the pancreatic β-cell regeneration program in the pancreatic tissue, but it is likely to act in combination with other factors to bring about a profound activation of Neuro D in the diabetic condition. The identity of the contributing factors is unknown. The other possibility is the involvement of Transforming growth factor-β signaling. Our Western blotting data show that the expression of Glut2 and Nkx6.1, the important pancreatic β-cell function markers, increased in the *db/db* group mice as compared to that in the non-diabetic groups, regardless of hydrolyzed SF treatment of the pancreatic tissue (Figure 4). The *db/db* mouse exhibits clinical and histological features of diabetic nephropathy that recapitulate the human disease, with an increase in the expression levels of TGF-β in the kidney. The advanced glycation end products (AGE) produced by the nonenzymatic reactions with glucose stimulate TGF-β production by the kidney cells, which then induces the expression of Glut2 and Nkx6.1 in the pancreas [31,32]. These data suggest that hyperglycemia or the other extrinsic damages, as well as the intrinsic damages, might induce the pancreatic β-cell regeneration signals. Hence, the role of hydrolyzed SF in regulating transcriptional factors requires more study. It will also be interesting to determine whether the treatment with hydrolyzed SF, or combining a 3D cell culture system with hydrolyzed SF as scaffold has any role in in vitro generation of mature pancreatic β-cells.

## 4. Materials and Methods

### 4.1. Preparation of Hydrolyzed Silk Fibroin

Hydrolyzed SF was purchased from Shindo Biosilk (Korea) and it was prepared by the degumming of raw cocoons (*Bombyx mori*, Chinese silkworm) with 5% Na_2_CO_3_ to remove sericin and other impurities. Pure SF was treated with 2N HCl aqueous solution at 100 °C for 48 h and neutralized using 2N NaOH. The solution was used to carry out proteolytic reactions using chymotrypsin (Sigma, St. Louis, MO, USA). The low molecular weight of SF was acquired through a desalting process using ion exchange chromatography.

### 4.2. Animals

We used C57BL/KsJ-*Lepr^db/db^* (*db/db*) and normal non-diabetic mice (C57BL/KsJ-*Lepr^db/+^* or *Lepr^+/+^*) that were supplied by SLC, Inc. (Shizuoka, Japan) at 6 weeks of age. Mice were given normal commercial laboratory chow (Jeiljedang, Seoul, Korea) and water ad libitum, except for mice that were given the 20% hydrolyzed SF solution instead of water. The *db/db* mice used in this study were 7 weeks of age, without distinction of sex (12). Animals were divided into the following 4 groups at 7 weeks of age: (1) normal non-diabetic mice (ND-NT group, *n* = 10) and (2) *db/db* mice (DB-NT group, *n* = 10), that were used as the control groups; (3) *db/db* mice (DB-T group, *n* = 10) and (4) normal non-diabetic mice (ND-T group, *n* = 10), that were provided with 20% drinking water and a mixture of hydrolyzed SF for 6 weeks. The animal study was conducted in accordance with the committee of laboratory animal care and use, Hallym University (Hallym 2013-132).

### 4.3. Measurement of Blood Glucose and Insulin Levels

Blood samples were collected from the tail vein (for glucose) or abdominal vein (for insulin) under anesthesia with isoflurane. The level of glucose was determined using the Accu-Check Performa (Roche, Copenhagen, Denmark). The insulin level was measured using an ELISA kit (Shibayagi, Gunma, Japan).

### 4.4. Pancreatic Islet Isolation

The mice were anesthetized with isoflurane, the peritoneum was surged, and the pancreas was removed after injecting collagenase P (Beohringer Mannheim, Germany) into the common the pancreatic duct. The extracted pancreas was incubated for 10 min at 37 °C and effused tissues from the digested pancreas were harvested. The harvested tissues were washed twice with Hank’s balanced salt solution (Gibco, Grand Island, NY, USA) by centrifugation, resuspended evenly in Ficoll (Sigma, St. Louis, MO, USA) with a density of 1.086 g/mL and overlaid with Ficoll at a density of 1.076 and 1.053 g/ml serially. Thereafter, the tube was centrifuged for 10 min at 800× *g* in a refrigerated centrifuge, and the islets present between the density of 1.076 and 1.053 were collected carefully, washed twice with HBSS, and the pellet was resuspended in M199 (Life Technologies, USA) containing 10% fetal bovine serum culture medium and incubated for 24 h in 5% CO_2_ incubator at 37 °C. The islets were purified manually under microscope. The purified islets were preserved in liquid nitrogen until subjected total RNA extraction.

### 4.5. Reverse Transcription—Polymerase Chain Reaction (RT-PCR)

Total RNA was extracted from the isolated islets using TRIzol Reagent (Life Technologies, USA). The first strand of cDNA synthesis by the reverse transcription of total RNA was performed by using a 1st Strand cDNA Synthesis Kit (Roche, Mannheim, Germany). Two micrograms of total RNA was used for cDNA reverse transcription with Superscript 3 (Life Technologies, Carlsbad, CA, USA). A total of 20 µL of each of these PCR products was separated on a 1% agarose gel in Tris–Borate–EDTA buffer (45 mM Tris–borate, 1 mM EDTA, pH 8.0) at 100 V and stained with ethidium bromide. Sequences for the primers used in this study are provided in Table S2.

### 4.6. Immunohistochemistry and In Situ Hybridization

After 6 weeks of experiment, the whole pancreatic tissue was resected from each mouse and weighed. These were fixed in 10% neutral buffered formalin, blocked in paraffin, and sectioned at 4 µm on an automatic rotary microtome (Zeiss, Jena, Germany). For immunofluorescence, the sections of the pancreatic portions were stained overnight with the target antibodies: anti-insulin (#sc-9168, Santa Cruz biotechnology, Dallas, TX, USA) and anti-glucagon (#SC-7779, Santa Cruz biotechnology, Dallas, TX, USA). Tissues were washed with phosphate buffered saline (PBS) and incubated with the secondary antibodies: anti-rabbit Alexa 594 (Life Technologies, Carlsbad, CA, USA) and anti-goat Alexa 488 (Life Technologies, Carlsbad, CA, USA). In the case of PCNA staining, tissues were incubated with the primary PCNA antibody (Vector lab, Burlingame, CA, USA) overnight. After washing with PBS, the sections were peroxidase-labeled using the Vector-statin ABC system (Vector lab, Burlingame, CA, USA), then developed with 3,3′-diaminobenzidine (DAB), and counterstained with Meyer’s hematoxylin. A BrdU assay was performed in accordance with manufacturer’s protocol (Sigma, USA). RNA in situ hybridization was performed as previously described [33].

### 4.7. Western Blot Analysis

The following antibodies were used: anti-insulin (#sc-9168, Santa Cruz biotechnology, USA), anti-Ngn3 (#SC-13793, Santa Cruz biotechnology, Dallas, TX, USA), anti-Neuro D (#SC-1084, Santa Cruz biotechnology, Dallas, TX, USA), anti-glucagon (#SC-13091, Santa Cruz biotechnology, Dallas, TX, USA), anti-Pax6 (#sc-11357, Santa Cruz biotechnology, Dallas, TX, USA), anti-Nkx2.2 (#sc-15013, Santa Cruz biotechnology, Dallas, TX, USA), anti-Nkx6.1 (#sc-15027, Santa Cruz biotechnology, Dallas, TX, USA), and anti-actin (A3854, Sigma, St. Louis, MO, USA). Western blotting was performed as previously described [34]. For quantification of the relative protein expression, the density of the protein band was measured using ImageJ software (NIH, Bethesda, MD, USA).

### 4.8. Statistical Analysis

Statistical analysis was performed using the GraphPad Prism 8 program (GraphPad software, San Diego, CA, USA). The results are presented as mean ± standard error of the mean (SEM), and the significance was set at *p* < 0.05. For comparison between two groups, the unpaired Student’s *t* test was used. For multiple comparisons, the one-way analysis of variance (ANOVA) was used.

## Supplementary Materials

The following are available online, supplementary information (Figure S1, Tables S1 and S2).
