# Supplementary material for: Silk Fibroin Promotes the Regeneration of Pancreatic β-Cells in the C57BL/KsJ-Leprdb/db Mouse
Source: Molecules. 2020 Jul 17;25(14):3259. doi: 10.3390/molecules25143259 (PMC7397053; doi:10.3390/molecules25143259)
Supplement: Supplementary file 1 [file molecules-25-03259-s001.pdf]

Supplementary Information for

Silk fibroin promotes the regeneration of pancreatic β-cells in the C57BL/KsJ-*Lepr*$^{db/db}$ mouse

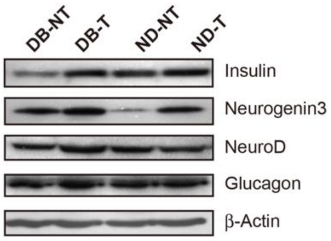

Figure. S1

Figure S1. Effects of hydrolyzed SF on the transcriptional factors in the isolated pancreatic islets. Western blot analysis of pancreatic islet with antibodies against insulin, Neurogenin 3, Neuro D, glucagon, and actin (DB-NT: *db/db* SF non-treated, DB-T: *db/db* SF treated, ND-NT: *db/+* SF non-treated, and ND-T: *db/+* SF treated).

Table S1. Effects of hydrolyzed SF on body weight

	ND-NT		ND-T		DB-NT		DB-T	
Time	0 week	6 weeks	0 week	6 weeks	0 week	6 weeks	0 week	6 weeks
Body weight	26.76 ±1.56	33.38 ±1.94	26.06 ±0.94	29.18 ±0.89	40.08 ±2.65	51.37 ±7.26	39.89 ±3.13	51.05 ±2.88

Data are presented as mean ± standard deviation (SD). There are no statistical differences in the start point or end of the experiment (ND-NT VS ND-T or DB-NT VS DB-T).

Table S2. Primers used for RT-PCR analysis

Primer Name	Sequence
Insulin-s	5′-TCT TCT ACA CAC CCA TGT CCC-3′
Insulin-as	5′-GGT GCA GCA CTG ATC CAC-3′
Somatostatin-s	5′-ATG CTG TCC TGC CGT CTC-3′
Somatostatin-as	5′-TTC TCT GTC TGG TTG GGC TC-3′
Gapdh-s	5′-AGG TCG GTG AAC GGA TTT C-3′
Gapdh-as	5′-TGT AGA CCA TGT AGT TGA GGT CA-3′

IGF-1-s	5′-GAA AAT CAG CAG TCT TCC AAC-3′
IGF-1-as	5′-TCC GGA AGC AAC ACT CAT-3′
EGF-s	5′-ATA GTT ATC CAG GAT GCC CAT-3′
EGF-as	5′-CGA TCC CCA GAA TAG CCA AT-3′
NeuroD-s	5′-AGT CAT GAG TGC CCA GCT TA-3′
NeuroD-as	5′-TTC TTC CAA AGG CAG TAA CG-3′
PDX-1-s	5′-AGA GGA CCC GAT CTG CCT AC-3′
PDX-1-as	5′-GGT CCC GCT ACT ACG TTT CT-3′
SP1-s	5′-AAG CTC TTC AGG CCC TCC-3′
SP1-as	5′-CTG CTG CTA GTC TGC CCC-3′
